# Structural Variation and Its Roles in Plant Genomes

**DOI:** 10.3390/plants15162498

**Published:** 2026-08-18

**Authors:** Ruyi Liu, Letong Huang, Jingru Mu, Ting Lu, Yifei Zhang, Kuanping Deng, Delin Xu

**Affiliations:** 1 Department of Medical Instrumental Analysis, Zunyi Medical University, Zunyi 563099, Chinazmuhlt0530@163.com (L.H.);; 2 Key Laboratory of Cancer Prevention and Treatment of Guizhou Province, Zunyi 563000, China; 3 Special Key Laboratory of Oral Disease Research and High Education Institute in Guizhou Province, School of Stomatology, Zunyi Medical University, Zunyi 563000, China; ashley16888@163.com; 4 Zunyi Academy of Agricultural Sciences, Zunyi 563099, China

**Keywords:** structural variation, plant genome, pan-genome, graph genome, long-read sequencing, multi-omics integration, crop breeding

## Abstract

Plant genomes exhibit extensive structural diversity generated by large-scale genomic alterations, collectively known as structural variations (SVs). Unlike single nucleotide polymorphisms (SNPs) and small insertions/deletions (indels), SVs can reshape genome architecture through changes in sequence content, gene dosage, regulatory landscapes, and chromosome organization. Recent advances in long-read sequencing (LRS), pan-genome construction, and multi-omics technologies have greatly expanded our ability to identify and interpret SVs across plant species. In this review, we summarize recent progress in understanding the formation mechanisms, classification, and functional consequences of plant SVs. We discuss major sources of SV generation, including transposable element activity, non-allelic homologous recombination (NAHR), horizontal gene transfer (HGT), and genome restructuring following polyploidization. We further highlight how LRS and graph-based pan-genomes overcome limitations of traditional linear reference genomes and enable more comprehensive characterization of genetic diversity. Beyond variant discovery, we emphasize the importance of integrating genomic, transcriptomic, epigenomic, proteomic, metabolomic, and spatial omics datasets to decipher how SVs influence gene regulation and complex agronomic traits. We also discuss current challenges, including repetitive genomes, polyploidy, computational complexity, and translation of SV knowledge into practical breeding applications. Together, these advances establish SV-centered genomics as a critical framework for understanding plant genome evolution and accelerating precision crop improvement.

## 1. Introduction

Plant genomics is a vital field within life sciences, dedicated to uncovering how genome organization, genetic variation, and regulatory mechanisms shape biological diversity and agronomic traits [1]. Its remarkable complexity, rich biological diversity, and profound agricultural significance have driven nearly half a century of technological innovation and theoretical exploration [2]. Since the publication of the first model plant genome, *Arabidopsis thaliana*, genome sequencing technologies have rapidly advanced, enabling comprehensive investigation of genome structure, evolution, and function [3,4]. Plant genomes contain both conserved genomic components shared among individuals and highly variable genomic regions that differ between populations or species. These variable regions include lineage-specific genes, Presence-absence variations (PAVs), structural variation (SV), and regulatory sequences that contribute to adaptation, environmental responses, and agronomic diversity [1,5,6]. Disease resistance genes (R genes) frequently exhibit extensive structural diversity in plants. Notably, major disease-resistance (R) genes typically feature a classic Nucleotide-Binding Site—Leucine-Rich Repeat (NBS-LRR) structure [7]; for instance, key SNPs often co-localize with NBS-LRR genes [8], playing a crucial role in biotic stress responses [9]. These R genes are predominantly distributed in rapidly evolving genomic regions and frequently undergo duplication, deletion, and rearrangement [10], providing abundant genetic resources for plant defense against pathogens [11]. Consistent with these findings, accumulating SV-focused studies further confirm that gene duplication may be linked to adaptation, and SVs can directly alter the expression levels of genes associated with agronomic traits [7]. Beyond small-scale sequence variations, increasing evidence demonstrates that SVs represent a major source of plant genomic diversity [12]. SVs can directly influence gene dosage, disrupt coding sequences, modify regulatory elements, and reshape chromatin organization, thereby driving phenotypic variation and evolutionary adaptation in plants [13].

As plant science enters the era of the “pan-genome” and the “3D genome” [14], researchers can now transcend the limitations of traditional reference genomes to comprehensively reveal the full spectrum of genetic variation [15] and the molecular logic underlying trait regulation at the species, population, and even single-cell levels [16]. Previously, it was widely accepted that most DNA sequences within a species are shared among individuals (the core genome), alongside abundant SNPs and small indels [17]. These common genetic variants have been extensively studied to elucidate the genetic basis of complex traits and to guide breeding practices [18,19]. However, recent studies have demonstrated that many DNA sequences are variable and shared only among a subset of individuals (the dispensable genome), giving rise to the novel concept of the “pan-genome” [20]. Defined as the complete collection of all DNA sequence diversity within a species [21], the pan-genome overcomes the constraints of traditional single linear reference genomes. By integrating all intra-species genetic variations (such as SNPs and SVs), it provides a more comprehensive and accurate characterization of plant genomes [22]. Soybean was the first crop to have its pan-genome assembled [22], and since then, pan-genomic research has expanded to numerous other plant species. In recent years, pan-genomics has evolved into a critical platform that integrates multi-dimensional data, systematically deciphers species diversity, and directly empowers breeding. For example, the construction of a sorghum pan-genome has revealed genetic variations and trait associations, providing a model for crop breeding [23,24].

Meanwhile, the 3D genome provides a spatial framework for understanding the SVs identified through pan-genomics [25]. The 3D genome refers to the spatial organization of chromatin within the nucleus, highlighting its critical role in gene regulation. This includes topologically associating domains (TADs) and the dynamic rearrangement of chromatin compartments, all of which are essential for understanding genome function [26,27,28]. The integration of pan-genomics and 3D genomics is expected to deepen our understanding of the structure-function relationships of plant genomes from both the sequence and spatial dimensions [29].

Since the year 2000, plant genome sequencing technologies have undergone a paradigm shift from low-throughput and high-cost to high-throughput and low-cost approaches [30]. LRS technologies, represented by PacBio HiFi and Oxford Nanopore, have significantly improved genome assembly contiguity, facilitating the resolution of SVs in complex genomes [31,32,33]. When integrated with techniques such as Hi-C and Chromatin Interaction Analysis by Paired-End Tag Sequencing (ChIA-PET), studies of 3D genome architecture have revealed the plasticity of chromatin organization. Environmental stresses or genetic perturbations can reshape chromatin structures and influence the expression of cryptic genetic variation [34], providing a foundation for understanding the role of SVs in genome dynamics.

SVs represent genetic differences among individuals and can lead to gene loss, duplication, or the emergence of novel genes, thereby driving phenotypic divergence [35]. Their formation is primarily driven by biological processes including transposable element (TE) activity [36], NAHR [37], HGT [38], and biased gene loss following polyploidization [39]. Among these, Transposable Elements (TEs) serve as the core drivers of genome plasticity [40]. Through mechanisms involving Class I retrotransposons, Class II DNA transposons, and Helitrons [41], TEs induce various SVs, such as indels, inversions, and duplicative expansions [42]. Furthermore, they drive lineage-specific genome expansion and rearrangement after species divergence [43,44]. TE-derived SVs play a crucial role in crop domestication [17]; for instance, TE insertions such as Hopscotch and miniature inverted-repeat transposable elements (MITEs) in maize regulate genes associated with branching and flowering time [45,46,47,48]. NAHR typically occurs in repetitive genomic regions, resulting in deletions, duplications, or inversions [37], and leads to inter-subgenomic structural differences in allopolyploid crops [39]. HGT introduces exogenous SVs through distant hybridization, involving mechanisms such as non-homologous recombination, TE activation, and chromosomal segment exchange [38,49,50,51]. Additionally, the diploidization process following polyploidization shapes SVs through biased gene loss and chromosomal rearrangements [52]. Collectively, these mechanisms highlight SVs—particularly those driven by TEs—as key drivers of genome plasticity, laying the groundwork for the subsequent discussion on their specific effects in crop domestication and agronomic applications.

The aim of this review is to provide a comprehensive overview of the recent advances in the study of plant genome structure and function [53]. We summarize current state-of-the-art sequencing methods for plant genomic analysis. Furthermore, we highlight the latest progress in understanding plant genomic variations and their applications in agronomy [54].

Compared with previously published reviews that often focus on individual aspects—such as pan-genome construction, SV detection algorithms, or multi-omics technologies separately—this review provides an integrated framework that explicitly links SV formation mechanisms, detection technologies, graph-based pan-genome representation, multi-layered functional interpretation, and breeding applications into a single coherent pipeline. In particular, we synthesize recent advances in plant 3D genomics, single-cell and spatial transcriptomics, and machine-learning-based SV prediction, which have not been systematically integrated in earlier reviews. By presenting a hierarchical workflow from genotype to phenotype, we aim to offer a practical roadmap for researchers seeking to translate SV discovery into actionable breeding strategies. Centered on SV as a connecting element between genome evolution and crop improvement, this review summarizes how SVs are generated, detected, represented within graph-based pan-genomes, and interpreted through multi-layer omics approaches, linking genomic variation with regulatory mechanisms, cellular processes, and breeding strategies to provide a comprehensive framework for understanding how structural diversity contributes to plant adaptation and agricultural innovation.

## 2. SVs in Plant Genomes: Types, Mechanisms, and Agronomic Impacts

SVs represent large genomic alterations generally larger than 50 bp, including deletions, insertions, duplications, inversions, translocations, and copy number variations (CNVs). Unlike SNPs and small indels, SVs can reshape genome architecture by modifying gene dosage, disrupting coding regions, altering regulatory landscapes, and generating novel gene combinations. Therefore, SVs constitute a major source of genetic diversity and play essential roles in plant adaptation, domestication, and trait evolution [12,13].

### 2.1. Classification and Biological Significance of SVs

The organizational architecture of eukaryotic genomes is divided into three hierarchical levels: primary structure (1D linear chromatin, encompassing DNA sequences, DNA-binding proteins, nucleosomes, etc.), secondary structure (local chromatin configurations formed by nucleosome interactions), and higher-order 3D structure (such as chromosome territories [CTs], A/B compartments, TADs, and chromatin loops) [55,56]. The 3D genome architecture is highly plastic [29]; environmental stresses or genetic perturbations can reshape chromatin organization, thereby influencing the expression of genetic variations. This plasticity highlights its potential role in crop domestication and adaptive evolution [34]. Within this hierarchical architecture, SVs are among the core drivers of genome plasticity and phenotypic divergence [57,58]. SVs refer to large-scale alterations in DNA sequences involving changes in length, copy number, orientation, or chromosomal position.

SVs can be categorized according to their distinct genomic consequences, and the major types include deletions, insertions, CNVs, inversions, and translocations [35,59]. Deletions remove genomic segments and may eliminate functional genes or regulatory elements. Insertions introduce additional sequences, including transposable element (TE)-derived fragments or foreign DNA sequences [60]. Duplications increase gene copy numbers and may contribute to gene family expansion and functional diversification. Inversions reverse the orientation of genomic regions and can suppress recombination, facilitating the maintenance of adaptive haplotypes. Translocations rearrange chromosomal segments and may generate novel regulatory environments [12]. Ubiquitously distributed in both nuclear and organellar genomes, these SV events can cause widespread gene gain or loss, gene duplication, and even the emergence of novel genes, thereby forming a crucial genetic basis for interspecific phenotypic diversity [61]. The typical manifestation of SV-induced gene turnover is reflected in lineage-specific structural differentiation of mitochondrial gene compositions across plant lineages [62]. For example, mitochondrial genomic comparisons among four Cyperaceae species have identified complete loss of sdh3 and specific pseudogenization of rps14 and rpl16 in water chestnut (*Eleocharis dulcis*), providing direct evidence of SV-mediated genomic divergence [63].

Compared with single-nucleotide polymorphisms (SNPs), SVs affect substantially larger genomic regions and therefore frequently produce stronger effects on gene expression and phenotypic variation [64]. Recent population-scale studies have demonstrated that SVs contribute significantly to diverse agronomically and ecologically important quantitative traits, including flowering time [65], plant architecture [66], disease resistance, and environmental adaptation [67].

### 2.2. Molecular Mechanisms Underlying SV Formation

#### 2.2.1. Transposable Element Activity

TEs are among the most important endogenous drivers of plant genome plasticity and represent one of the most active sources of endogenous variation in plant genomes. According to their replication mechanisms, TEs are broadly classified into Class I retrotransposons and Class II DNA transposons. Class I retrotransposons propagate through an RNA intermediate via a “copy-and-paste” mechanism, while Class II DNA transposons generally move through a transposase-mediated “cut-and-paste” mechanism. TE activity involves both autonomous and non-autonomous elements; autonomous elements encode the enzymatic machinery required for their own mobilization, whereas non-autonomous elements lack complete coding capacity and rely entirely on autonomous partners for transposition [68,69,70]. Both can cause sequence indels, break repair, or local rearrangements at the insertion sites. Furthermore, Helitrons can capture and mobilize gene fragments, thereby facilitating gene duplication and the formation of novel chimeric structures [41,42,71,72].

Through insertion, deletion, amplification, and recombination, TE activity contributes substantially to genome expansion and structural diversification. When TEs insert into promoters, enhancers, or introns, they can alter the spacing of cis-regulatory elements [73], chromatin states, and transcript structures, further translating SVs into selectable expression differences [74]. For example, The Hopscotch insertion upstream of tb1 and the noncoding variation at the Vgt1 locus in maize, which regulate branching and flowering, respectively, demonstrate that TE-mediated SVs serve not only as a driving force for genome expansion but also as a crucial molecular basis for domestication traits [45,46].

Beyond maize, TE-derived SVs have also been associated with agronomic traits in rice [75], tomato [42], and wheat [76], indicating that TE-mediated genome remodeling is a widespread evolutionary mechanism rather than a species-specific phenomenon.

#### 2.2.2. NAHR and Related Recombination Events

NAHR occurs between highly similar but non-allelic repetitive sequences [77]. When repetitive copies misalign during meiosis or DNA double-strand break repair, crossing over can generate deletions, duplications, inversions, and complex rearrangements [78]. The breakpoints of these SVs are typically enriched in low-copy repeats, tandem repeats, or transposable element-associated regions [37]. In allopolyploid plants, the abundance of homoeologous segments among subgenomes means that NAHR, coupled with subsequent chromosomal rearrangements, amplifies copy number and synteny differences, thereby providing raw material for the retention or loss of adaptive genes [39]. Therefore, deciphering the composition of repetitive sequences and the microhomology at SV breakpoints is crucial for elucidating NAHR mechanisms and assessing their functional consequences [79].

NAHR should be strictly distinguished from two frequently confused recombination modes: unequal crossing over and ectopic recombination. Although NAHR and unequal crossing over both involve homologous sequence misalignment [80], unequal crossing over usually occurs during meiosis between mispaired homologous chromosomes or chromatids and primarily produces reciprocal duplication and deletion products [78]. In contrast, NAHR refers more broadly to recombination between non-allelic but homologous genomic regions [81,82] and can occur in both meiotic and mitotic contexts [78]. Another related mechanism is ectopic recombination, which involves recombination between repeated sequences located at abnormal genomic positions. Unlike canonical allelic recombination occurring between corresponding loci, ectopic recombination can trigger extensive chromosome rearrangements and profoundly contribute to genome instability [83].

#### 2.2.3. HGT and Genome Innovation

HGT introduces DNA fragments from distantly related species, parasitic plants, microbes, or viruses into recipient genomes, serving as a source of SV that transcends the boundaries of vertical inheritance [84]. Following integration via non-homologous end joining (NHEJ), homologous recombination, or transposon-mediated mechanisms, exogenous sequences may be retained as intact genes or gene clusters, or they may induce insertions, duplications, and rearrangements of adjacent sequences [38]. Comparative genomic studies in the Scenedesmaceae family have identified HGT-derived gene families with diverse origins that are associated with stress adaptation pathways, suggesting that TEs and endogenous viral elements may be involved in their mobilization or fixation [85].

Notably, distinguishing genuine HGT events from alternative interfering factors—including sequencing contamination, ancestral genetic polymorphism, and differential gene loss across lineages [86]—requires comprehensive integration of phylogenetic incongruence [87,88], sequencing read coverage, and genomic synteny evidence [89]. In addition, HGT should not be equated with distant hybridization. Distant hybridization relies on sexual reproduction between evolutionarily divergent species, whereas HGT enables direct acquisition of exogenous DNA independent of normal reproductive processes [90,91]. Although both pathways can introduce foreign genetic material into plant genomes, their underlying mechanisms and downstream genomic consequences are distinctly different.

HGT is not strictly limited to distantly related species; it can also occur between closely related species or even within species through various mechanisms, though such events are less frequently documented and harder to distinguish from horizontal introgression.

#### 2.2.4. Polyploidization and Biased Gene Loss

Whole-genome duplication (WGD) acts as a pivotal source of structural genome variation, which generates a massive number of homoeologous or heterologous chromosomal and gene copies within a short timeframe, providing a highly redundant genomic background for the continuous accumulation of SVs [92]. Following WGD, plants undergo a long-term diploidization process, during which the genome gradually restores genetic stability through multiple regulatory pathways, including large-scale chromosomal rearrangements [93], asymmetric segment fractionation, transposable element reactivation [94], and intergenic gene conversion [95].

During diploidization, different subgenomes frequently exhibit unbalanced genetic performance, including biased gene retention, expression dominance, and functional divergence, a typical genomic phenomenon defined as biased gene loss or subgenome fractionation [39,52,96]. This process is far from random simple sequence deletion. Instead, genomes selectively retain dosage-sensitive genes, stress-responsive genes, and developmentally regulated functional genes while removing redundant sequences [97], which profoundly reshapes gene regulatory networks and enhances plant trait plasticity [98]. Consequently, polyploidization and subsequent biased gene loss constitute a crucial genetic foundation [99] for the adaptive evolution and agronomic improvement of polyploid crops.

### 2.3. Functional Consequences of SVs in Plant Evolution and Breeding

SVs influence plant phenotypes through multiple mechanisms, including gene dosage alteration, disruption or creation of regulatory elements, modification of chromatin organization [100], and generation of novel gene combinations. Recent advances in pan-genome analysis have revealed that many agronomically important genes are located within variable genomic regions rather than conserved core regions. These findings highlight the importance of incorporating SV information into crop improvement strategies [101]. As pivotal drivers of genome plasticity, species evolution, and the formation of key agronomic traits, SVs shape plant adaptation, domestication, and breeding potential throughout plant growth and evolution [42]. Their formation primarily stems from core biological processes, including TE activity, NAHR, HGT, and biased gene loss following polyploidization [44,45,46,47]. As detailed in Section 2.2, TEs drive genome expansion and structural diversification through insertion, amplification, and recombination; representative functional consequences are described below.

TE-derived SVs have played a crucial role in crop domestication. A series of classical functional studies have confirmed their mechanism of altering gene expression as cis-regulatory elements and driving novel phenotypes [17]. Taking maize as an example: the insertion of the Hopscotch transposon 62 kb upstream of the tb1 gene upregulates its expression via an enhancer effect, thereby suppressing lateral branch growth, which is recognized as one of the key events in maize domestication [46]; the presence of a MITE transposon at the Vgt1 locus significantly shortens flowering time by regulating ZmRap2.7 expression [45]; and the insertion of Harbinger-like and CACTA transposons in the promoter regions of ZmCCT9 and ZmCCT10 modulates photoperiod sensitivity through epigenetic silencing mechanisms [47,48]. These cases illustrate that TE-derived SVs constitute an essential genetic basis for crop domestication and improvement. Similarly, in the ornamental plant foxglove (*Digitalis purpurea*), the white-flower phenotype results from the insertion of a ~14.2 kb transposon within the anthocyanin synthase gene (ANS). This insertion disrupts the normal transcriptional structure of the gene, impeding anthocyanin biosynthesis, thereby elucidating the molecular basis of flower color variation [102]. These representative cases demonstrate that TE-derived SVs are the essential genetic basis for crop domestication and trait improvement.

In addition to TEs, NAHR is another critical mechanism for SV formation. It typically occurs in repetitive sequence regions, resulting in deletions, duplications, or inversions, which is particularly prominent in the subgenome differentiation of allopolyploid wheat [37,39]. Distinct from NAHR, HGT introduces exogenous SVs through distant hybridization, involving processes such as non-homologous recombination and TE activation [38]. This can be categorized into three types: (1) Non-homologous recombination: Distant hybridization may compromise genome stability, triggering NHEJ or replication fork stalling, leading to segmental duplications or deletions [49,50]. (2) Transposon activation: Hybridization stress may activate TEs (e.g., the Starship family), driving the mobilization or amplification of large DNA fragments to form CNVs [51]. (3) Chromosomal segment exchange: Following distant hybridization, incomplete pairing between parental genomes may induce chromosome breakage-fusion events, generating subtelomeric rearrangements (e.g., CNVs of the EPSPS gene in Elymus species) [49].

Additionally, the diploidization process following polyploidization continuously shapes genome architecture through biased gene loss and chromosomal rearrangements [52]. Research on genomic variation has been extensively applied in agronomy. SVs not only drive the aforementioned key domestication traits but also broadly shape agronomic traits, genome architecture, and adaptive evolution in various crops [24,103]. For instance, in Solanaceae crops, centromeric regions are composed of specific Long Terminal Repeat (LTR) retrotransposons, and inversions surrounding these regions drive centromere repositioning, serving as a key mechanism for species divergence [104]. Table 1 highlights representative SV studies in model plants and major crops in recent years, covering aspects ranging from classical domestication traits (e.g., plant architecture, flowering time, quality) to complex agronomic traits (e.g., heterosis, environmental adaptation). This aims to demonstrate the diversity and importance of SVs across different plant taxa and functional dimensions.

With the rapid development of pan-genomics, SV research has advanced from traditional single-gene mapping to multi-dimensional integrative analysis, greatly improving the application potential of SVs in precision crop breeding. Pan-genome, PAV, and population genetic analyses have been widely combined to dissect complex agronomic traits. For example, pan-genome-based SV analysis in wax apple successfully identified causal genes controlling fruit size and cold tolerance [113]. In pepper, Jasmonate ZIM-domain proteins (*JAZs*) and truncated conserved Proline-Tyrosine (P-Y) anchors reduce Methyl Jasmonate (MeJA) -triggered degradation, forming molecular brakes. Two haplotypes provide genetic targets balancing growth and defense for climate-adaptive pepper breeding [114].

Recent advances in pan-genome analysis have further revealed that most agronomically critical genes reside in variable genomic regions rather than conserved core genomes. However, genes governing fundamental developmental processes often remain highly conserved [115]. Consistent with previous findings, functionally vital SVs associated with disease-resistance clusters, flowering regulatory pathways, and metabolic diversification have been extensively identified in major crops, including rice (*Oryza sativa*) [116], tomato (*Solanum lycopersicum*) [117], pepper (*Capsicum annuum*) [118], sorghum (*Sorghum bicolor*), and wheat (*Triticum aestivum*) [119]. Accordingly, SVs should no longer be regarded as trivial, passive genomic differences. Instead, they represent core functional genetic components that profoundly shape plant adaptation, domestication, and modern crop breeding potential [24,120].

## 3. Technologies Enabling SV Discovery and Functional Interpretation

The identification and interpretation of SVs require a combination of complementary technologies rather than a single sequencing platform. Recent advances have transformed plant genomics from linear reference-based analysis toward population-scale pan-genome construction and multi-layer functional interpretation.

A complete SV analysis framework generally involves four interconnected stages: (1) accurate genome assembly and SV detection; (2) representation of genetic diversity through pan-genomes or graph genomes; (3) integration with regulatory and molecular datasets for functional interpretation; and (4) experimental validation and breeding application.

### 3.1. Evolution of Sequencing Technologies for SV Detection

#### 3.1.1. Short-Read Sequencing: High Accuracy but Limited Resolution

Illumina short-read sequencing has played a foundational role in plant genomics due to its high accuracy, low cost, and scalability [121]. It has been widely applied in population resequencing, genome-wide association studies (GWAS), and transcriptome profiling [121,122].

Like classic Next-Generation Sequencing (NGS), Illumina relies on Sequencing-By-Synthesis (SBS) base calling via fluorescent signals. With >99.9% accuracy, 2–750 Gb per run, and low cost [123], it serves large-scale resequencing, variant calling, QTL mapping, and RNA Sequencing (RNA-seq) [124,125]. Short-read-based reduced-representation sequencing can also efficiently develop abundant SNPs for population genetics research [126,127].

Nevertheless, short reads of only hundreds of base pairs fail to span repetitive genomic regions, restricting reliable detection of large SVs using short fragments. This limitation is particularly severe in plant genomes enriched with TEs, segmental duplications, and highly repetitive heterochromatic regions. Accordingly, short-read sequencing remains valuable for population-scale genotyping but is insufficient alone for comprehensive SV discovery [128].

#### 3.1.2. LRS: Resolving Complex Plant Genomes

LRS technologies, including PacBio HiFi sequencing and Oxford Nanopore Technologies (ONT), have significantly improved the resolution of plant genome assemblies and revolutionized the dissection of complex plant genomes [129]. LRS, also known as third-generation sequencing (TGS), generates reads ranging from kb to Mb, outperforming short reads [130,131]. It resolves repeats and complex SVs, including haplotyping polyploids [131].

PacBio HiFi sequencing leverages Single Molecule, Real-Time (SMRT) technology with polymerase fluorescence detection to generate long reads, then applies circular consensus sequencing (CCS) to achieve >Q20 accuracy—enabling chromosome-scale assemblies with precise haplotype resolution [132,133]. Optimized low-coverage HiFi pipelines facilitate SV calling in polyploid wheat [134]. It has successfully assembled the 78.64% repeat-rich, highly heterozygous *Manglietia pachyphylla* genome [135], which is intractable for short-read platforms.

ONT detects current shifts as nucleic acids pass nanopores, producing ~300 kb ultra-long reads often combined with Illumina data [136]. Portable devices support direct sequencing for complex assembly and real-time analysis [137]. ONT plus trio-binning phased two parental haplotypes of hybrid *Eucalyptus*, uncovering SVs underlying heterosis [108].

Compared with short-read sequencing, long-read approaches directly span repetitive sequences and SV breakpoints, enabling more accurate detection of insertions, inversions, translocations, and PAVs. For example, LRS has enabled the construction of highly contiguous genomes for complex plant species, including notoginseng (*Panax notoginseng*) [138], and multiple polyploid crops [139,140]. These examples were selected because these species contain highly repetitive genomes and complex structural organization, representing typical challenges that cannot be fully resolved using short-read approaches [141]. Short-read assemblies often result in assembly collapse, compressing multi-member tandem arrays into a single consensus sequence, whereas long-read-based pangenome approaches restore tandem arrays as discoverable, measurable objects [141].

Combined multi-sequencing strategies further tackle intricate plant genomes. The integration of PacBio HiFi and Hi-C data enables telomere-to-telomere (T2T) gap-free genome assembly for wheat, *Eucalyptus*, and alpine medicinal plant *Rhodiola yunnanensis* [142], providing high-quality genomic resources for high-altitude adaptation mechanism research [143]. The combination of ONT long reads and Illumina short reads corrects base errors and assembles the high-quality genome of Mowanggu rice, and successfully identifies blast-resistance-related SV at the Pi49 locus [144]. Overall, integrated long-read and multi-omics data drive modern plant genomics toward high-accuracy, gap-free, and multi-dimensional systematic research.

#### 3.1.3. Hi-C and Three-Dimensional Genome Technologies

Although sequencing technologies reveal linear DNA genome sequences, SV interpretation increasingly requires understanding their regulatory effects on three-dimensional genome organization [14]. Hi-C and related chromatin interaction technologies uncover chromatin compartmentalization, topological domains, and long-range chromatin interaction relationships [145], covering core 3D genome architectures including CTs, A/B compartments, TADs, and enhancer–promoter interactions [146].

Integration of Hi-C with long-read assemblies allows researchers to determine whether structural rearrangements modify regulatory landscapes rather than simply alter DNA sequences [147]. Assay for Transposase-Accessible Chromatin using sequencing (ATAC-seq), Chromatin Immunoprecipitation Sequencing (ChIP-seq) and Bisulfite Sequencing (BS-seq) technologies further characterize chromatin accessibility, histone modifications and DNA methylation status at the genome-wide level. Integrating these multi-layered 3D genomic and epigenetic data with transcriptomics enables systematic interpretation of gene regulation via the complete cascade: “genetic variant-chromatin conformation-epigenetic state-gene expression” [148,149].

Gains or losses of enhancer-promoter interactions induced by SVs are usually accompanied by altered local open chromatin status and changed activating/repressive epigenetic marks, ultimately modulating target gene transcription and phenotypic formation [150]. SVs can disrupt or establish new spatial contacts between enhancers and promoters, which directly affects the loosening or compaction of local chromatin structure. The dynamic changes in chromatin accessibility further alter the enrichment levels of histone activation modifications (such as H3K4me3 and H3K36me3) and repressive modifications (such as H3K27me3), thereby switching the transcriptional activation or silencing state of target genes and driving the generation of distinct biological phenotypes [149,151,152,153]. In crop domestication, structural variants (SVs) not only alter gene dosage or cis-regulatory elements at the linear genome level, but also reshape distal regulatory contacts by rearranging chromatin neighborhoods and rewiring 3D regulatory networks [154]. Different from static linear genomic variations, SV-mediated 3D chromatin rearrangement can break the original regulatory topological boundaries, enable long-distance non-coding regulatory elements to interact with target genes, or isolate genes from their original regulatory modules, which greatly enriches the regulatory diversity of key domestication genes and provides an abundant genetic variation basis for the selection and improvement of crop agronomic traits [154,155]. For instance, chromosomal inversions may suppress meiotic recombination by reorganizing local chromosomal regions, whereas large insertions may introduce novel regulatory elements or alter chromatin accessibility, thereby driving phenotypic variation [156]. Accordingly, parallel comparisons of SV breakpoints, Hi-C interaction patterns, epigenetic signatures and gene expression profiles should be performed on identical genetic materials, followed by causal functional validation through reporter assays, gene editing or complementation tests.

### 3.2. Graph Pan-Genomes: Beyond Linear Reference Genomes

Traditional plant genome analyses rely mainly on a single linear reference genome. Although effective for identifying variants close to the reference sequence, this approach introduces severe reference bias because novel sequences and structural variants absent from the reference genome cannot be efficiently detected or accurately genotyped [57].

Graph pan-genomes overcome this limitation by representing genomic diversity as interconnected alternative paths rather than a single linear sequence. In a graph genome, multiple haplotypes, insertions, deletions, and structural variants can be simultaneously represented, allowing more accurate alignment and genotyping across diverse plant populations [157].

#### 3.2.1. Advantages of Graph Pan-Genomes for SV Detection

Compared with linear reference genomes, graph pan-genomes provide three major advantages for plant SV detection and genetic diversity analysis [158]:

- Reduction in Reference Bias: In linear-reference analysis, sequencing reads originating from highly divergent genomic regions may fail to align or may be incorrectly mapped, leading to missing or false variant calls. Graph genome representations fully incorporate alternative non-reference sequences, effectively improving the detection accuracy of population-specific structural variants and rare genetic variations [159].
- Improved Detection of Insertions and Presence–absence Variations: Large insertions and PAVs represent some of the most difficult variant types for linear-reference approaches because the inserted sequences are completely absent from the reference genome. Graph genomes directly encode alternative genetic paths, enabling systematic identification of novel genes or regulatory regions present only in subsets of individual plant accessions [160].
- Better Genotyping of Complex Structural Variants: Many functional SVs in plants are not simple deletions or insertions but involve complex combinations of multiple chromosomal rearrangements. Graph-based approaches allow complex haplotypes and nested SVs to be represented and compared simultaneously, substantially improving population-scale genotyping accuracy for complex SV loci [161].

Mechanistically, graph pan-genomes reduce reference bias by encoding alternative alleles as branched paths in a variation graph. When sequencing reads are aligned to this graph structure rather than a single linear sequence, reads originating from non-reference haplotypes can still be mapped accurately because the graph explicitly represents the alternative sequence as a valid path [162]. This contrasts with linear-reference alignment, where such reads would either be mismapped to the closest homologous region or discarded as unmapped. For complex SVs involving nested insertions, inversions, or translocations, graph genomes decompose these variants into connected subgraphs that preserve the relative order and orientation of rearranged segments, enabling simultaneous genotyping of multiple overlapping SVs that would be impossible to resolve using a single linear coordinate system [163].

#### 3.2.2. Applications of Graph Pan-Genomes in Crops

Graph pan-genomes have revealed substantial hidden genetic diversity and functional SV resources in major crops that are inaccessible via linear reference genomes.

In tomato (*Solanum lycopersicum*), a graph pangenome constructed from 838 tomato accessions successfully recovers structural variants missed by linear reference-based GWAS, and accurately quantifies their heritable contributions to key agronomic traits including fruit quality and disease resistance [141]. In *Capsicum*, pan-genome analysis integrating 16 genomes across three species complexes revealed that merely ~13% of the total gene set are core genes, with the remaining 87% forming the variable genome (shell and cloud genes), which are significantly enriched in disease-resistance and stress-adaptation pathways [118,164,165]. In soybean (*Glycine max*) and other major crops, pan-genome approaches have confirmed that variable genomic regions contain numerous functional genes associated with disease resistance [166], stress tolerance, and metabolic trait diversification [167].

Beyond model crops, graph pan-genomes also show powerful application potential in non-model plant species. The graph pangenome of *Acer palmatum* systematically characterizes millions of trait-linked SNPs and structural variants [168], and precisely identifies adaptive loci carrying tandem-array CNVs [169]. In research on plant-pathogen interaction mechanisms, ecological and evolutionary analyses of plant viral populations under graph genome regulatory frameworks further help elucidate how genomic variants drive viral adaptability and host resistance breakdown [170]. These findings indicate that graph pan-genomes provide an essential framework for discovering and mining breeding-relevant genetic resources that are missed by conventional reference-based genomic methods.

### 3.3. Integrated Computational Workflow for SV Discovery

**Figure 1:**
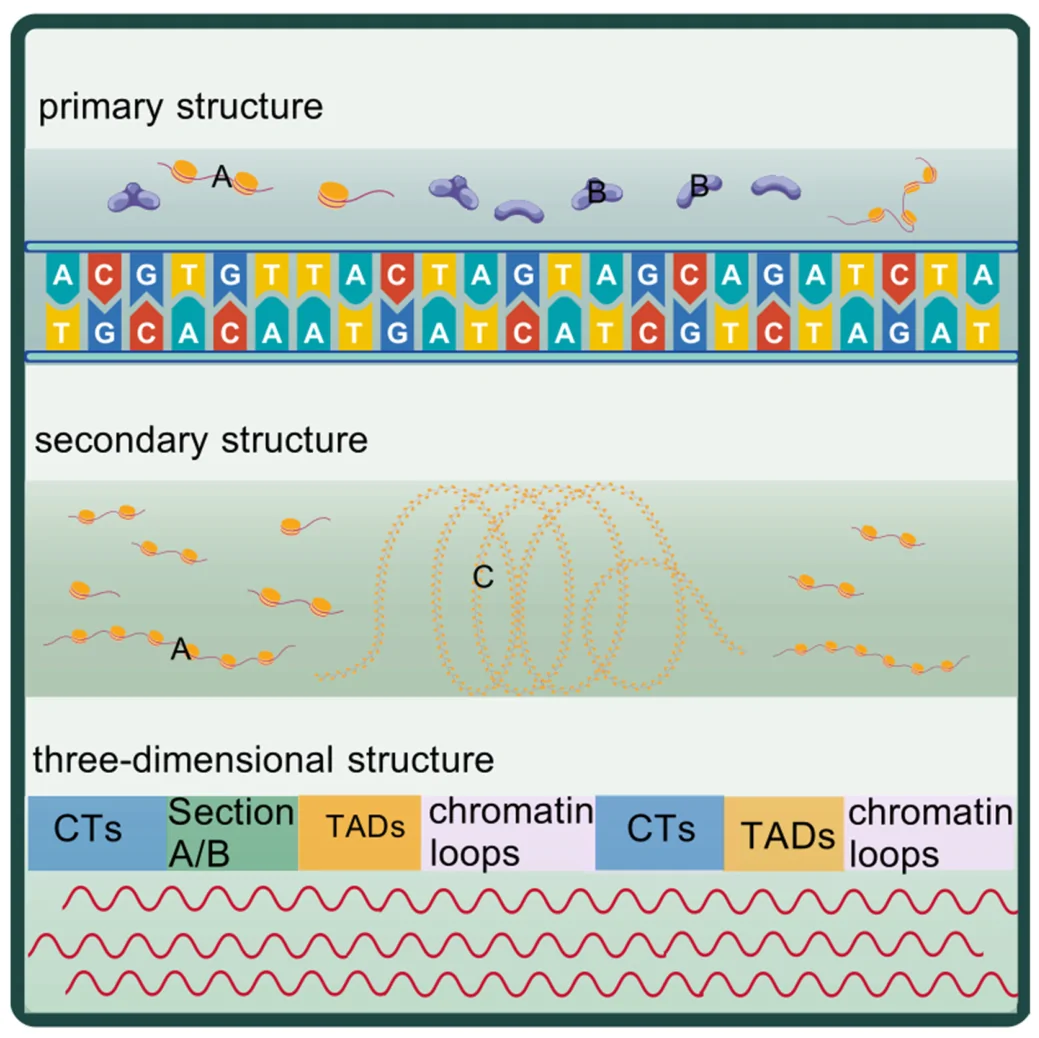
A simplified schematic diagram of genomic stratification. A. nucleosome. B. DNA-binding proteins. C. The local chromatin structures formed by interactions between nucleosomes. Hierarchical organization of plant genome architecture. The schematic illustrates the multi-level structural organization of plant genomes, from primary linear sequences (DNA and nucleosomes, A) through DNA-binding proteins (B) and local chromatin configurations (C), to higher-order three-dimensional folding (chromosome territories, A/B compartments, TADs, and chromatin loops). This hierarchical architecture provides the structural framework within which structural variations (SVs) can exert their regulatory effects by altering chromatin organization and gene expression. TAD, topologically associating domain.

The increasing complexity of plant genomes enriched with repetitive sequences and SVs requires integrated computational pipelines combining multiple sequencing and analytical approaches [171]. Given that genomic variants manifest across hierarchical chromatin architectures from linear sequences to 3D folding (Figure 1), bioinformatic tools act as a critical bridge linking raw sequencing data to biological discoveries. This section centers on core computational approaches and toolkits for deciphering plant genomic variants, especially structural variants (SVs).

**Figure 2:**
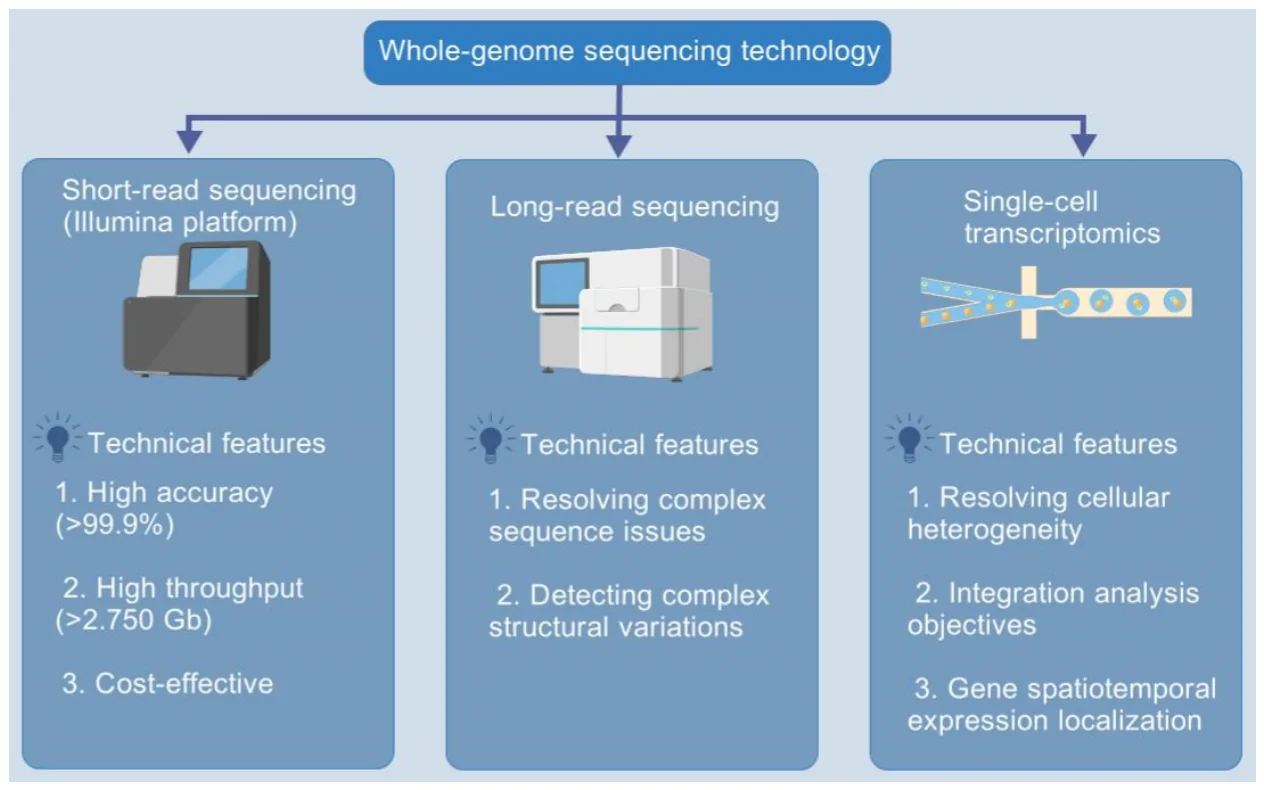
Plant genome sequencing technologies. Multi-omics framework for interpreting functional consequences of plant structural variations. Structural variations may influence phenotypes through multiple regulatory layers, including chromatin remodeling, altered transcription, protein abundance changes, metabolic pathway regulation, and cell-type-specific expression patterns. Integration of multi-dimensional datasets enables transition from association analysis toward causal understanding.

A complete and typical SV analytical workflow includes five interconnected steps:

1. Genome Assembly: LRS and Hi-C technologies are integrated to generate high-quality chromosome-scale assemblies and resolve complex repetitive regions and heterozygous sequences. As a haplotype-aware assembler, hifiasm retains complete haplotype structures from PacBio HiFi reads and outputs accurately phased genome assemblies. It perfectly preserves genetic divergence in tandem gene arrays, which is critical for resolving structural variants of tandemly duplicated genes [85]. Besides hifiasm, Flye is specifically optimized for continuous long reads such as Nanopore data, while NextDenovo supports multiple sequencing data types and is widely used for complex plant genome assembly. For research projects with high-quality reference genomes of closely related species, tools like Ragout 2 can anchor de novo assembled contigs to chromosomes via synteny analysis, representing a cost-effective assembly strategy [172]. After assembly, minimap2, the prevailing high-performance aligner, leverages minimizer indexing and chunked chaining algorithms to process error-prone long reads efficiently and accurately, with drastically improved speed compared with earlier tools, laying a solid foundation for subsequent variant calling [173]. Key technical features of these core assembly and alignment platforms are illustrated in Figure 2.

2. SV Identification: Multiple approaches, including read alignment, assembly comparison, and graph-based analysis, are combined to comprehensively detect deletions, insertions, CNVs, inversions, and translocations [35].
  Traditional short-read-based SV calling tools, including DELLY, Manta, and Lumpy, infer structural variants indirectly by analyzing read pairs, split reads, and read depth signals, yet they have inherent limitations in resolving complex repetitive regions and large-scale SVs [174]. In contrast, long reads can span variant regions directly, revolutionizing the accuracy and sensitivity of plant SV detection. Popular long-read SV callers include Sniffles2, cuteSV, pbsv and SVIM. Systematic benchmark tests demonstrate that Sniffles2 achieves the best overall F1-score for SV detection, whereas cuteSV yields a superior recall rate [175]. Advanced algorithm upgrades in Sniffles2 enable higher indel detection sensitivity on Nanopore data than traditional Illumina short reads [176].
  To further improve detection accuracy, ensemble strategies and machine learning are widely applied in SV calling. Outputs from distinct SV callers often show inconsistent and conflicting results. Ensemble pipelines such as SV-Meca [177] and VISTA [178] integrate prediction results and supporting evidence from multiple tools via machine learning frameworks (e.g., eXtreme Gradient Boosting [XGBoost]), assign credible confidence scores to each variant, efficiently reduce false positives and improve overall F1-scores, offering novel strategies for precise filtering of plant SVs. Filtered high-confidence structural variants can be further subjected to selection pressure analysis to dissect their evolutionary impacts. For instance, coding variants of *CaJAZ4* and *CaJAZ7* in the pepper *JAZ* family are driven by strong directional selection and participate in the long-term evolutionary arms races between plants and pathogens [114].

3. Pan-genome Construction: Detected high-confidence variants are incorporated into graph-based genome representations to comprehensively capture population-level genetic diversity. A complete plant pangenome consists of core genomes shared by all individuals of a species and variable genomes unique to subsets of individuals. Major tools for graph pangenome construction and analysis include minigraph (for rapid graph construction incorporating large structural variants), pggb (a fully automated pipeline for graph building, sequence alignment and haplotype ordering), and vg (a versatile variant graph toolkit with comprehensive graph manipulation algorithms) [179,180,181], which jointly support high-efficiency and high-accuracy graph pan-genome analysis in plants.
4. Functional Interpretation: SVs are integrated with transcriptomics, epigenomics, proteomics, metabolomics, and spatial transcriptomics datasets [182] to determine their regulatory mechanisms and phenotypic consequences. Multi-omics integration in plants faces unique challenges compared with animal or microbial systems, including widespread polyploidy, highly repetitive genomes, tissue heterogeneity, and strong environmental plasticity. Despite these obstacles, integrating multi-layer molecular data provides an effective strategy to link SVs to gene regulation and phenotypic variation [168,183].
  Multi-omics integration systematically dissects gene-to-phenotype regulatory networks by combining multi-layer molecular data [184,185]. Genomic and transcriptomic integration uses SNPs, structural variants (SVs) and presence–absence variants derived from population resequencing, pan-genomes or haplotype-resolved assemblies as genetic anchors to build joint analytical models with RNA-seq datasets from diverse tissues, developmental stages or treatments [186,187]. Expression quantitative trait locus (eQTL) analysis distinguishes cis- and trans-regulatory loci and links transcriptional variation to complex agronomic traits. In hybrids and polyploids, allele-specific expression (ASE) directly detects parental expression bias, providing direct molecular evidence for functional cis-regulatory variants [188]. This strategy is particularly powerful for dissecting functional non-coding SVs [189]. For instance, genomic data first identified the classic Hopscotch insertion upstream of maize tb1 and variants in the Vgt1 region [190]. Subsequent transcriptional divergence and tissue-specific expression profiles fully validated their regulatory effects on neighboring genes and domestication traits [45,46].
  A key principle is that deepening the functional interpretation of structural variants requires multi-omics synergy and should not depend on transcriptomics alone. Transcriptomics (ASE/eQTL) acts as the core; epigenomics and 3D chromatin data assist in interpreting non-coding SVs; proteomics and metabolomics validate functional translation; and spatial transcriptomics complements cell-specific information. A streamlined workflow can be proposed as follows: SV mapping → eQTL-based association → proteome- and metabolome-guided filtering → spatial validation, aided by weighted gene co-expression network analysis (WGCNA) and machine learning. Given the unique plant-specific challenges such as polyploidy and repetitive sequences, dedicated analytical workflows are necessary. Ultimately, cross-omics triangulation effectively removes false-positive signals, reinforces causal inference, and prioritizes key candidate SVs.
  Single-cell and spatial transcriptomics (Stereo-seq) have revolutionized our understanding of cellular heterogeneity and spatiotemporal gene expression regulation in plants [101]. It precisely locates transcripts at high spatial resolution, overcoming spatial information loss in standard bulk RNA-seq, and has been widely applied in plant development and stress biology research [102,103]. Stereo-seq has uncovered numerous key plant biological processes and trait regulatory mechanisms (Table 2). In Populus bolleana leaves, snRNA-seq combined with high-resolution spatial transcriptomics successfully generated a comprehensive cell atlas, revealing distinct transcriptional polarity along the adaxial–abaxial axis in phenylpropanoid biosynthesis, flavonoid metabolism, stress response and hormone signaling pathways, and identifying MYC2 as the core key regulator of asymmetric leaf cuticle formation [191,192,193,194]. These single-cell and spatial multi-omics studies reveal the cellular basis of major agronomic traits at high resolution and stand at the forefront for deciphering complex plant-microbiome interaction networks at cellular scale [195].
  Proteomic and metabolomic integration further complements transcriptional regulation research. Quantitative proteomics reflects post-transcriptional regulatory events including protein translation, modification and degradation, while metabolomics directly captures small-molecule end products and dynamic metabolic flux changes. Combined with transcriptomic data, they effectively distinguish whether transcriptional alterations are finally translated into functional phenotypic outputs and pinpoint key biochemical bottlenecks underlying trait formation. Notably, multi-omics integration should go beyond simple expression level comparison. At the organellar level, numerous RNA editing sites cluster in protein-coding regions of water chestnut mitochondrial transcripts, most of which alter amino acid hydrophobicity, indicating that post-transcriptional layers such as RNA editing, post-translational modifications, and protein turnover should be integrated into plant multi-omics analytical systems.
  The ultimate goal of multi-omics integration is to convert discrete molecular measurements into testable regulatory networks and transferable predictive models. In practice, co-expression networks, QTL co-localization analysis, Bayesian networks or multi-view machine learning can be adopted to integrate genomic, epigenomic, transcriptomic, proteomic and metabolomic data together with phenotypic and environmental information into a unified analytical framework. Key molecular hubs identified therefrom need further validation using independent populations and functional assays to prevent confusing correlation with causation [186,187]. Such models enable prioritized screening of regulatory genes and molecular modules governing complex traits, and facilitate molecular design breeding when combined with multi-environment phenotypic data. The cell-resolution atlas of *Populus bolleana* leaves exemplifies this systematic research paradigm [203].
  Standardized bioinformatic pipelines support the above multi-omics data analysis. For single-cell transcriptomic data, preprocessing is commonly performed via official pipelines (e.g., Cell Ranger from 10X Genomics) or universal alignment software STARsolo. Downstream analysis and visualization are dominated by two mainstream frameworks: Seurat (v4.0 or v5) in R [204] and Scanpy (v1.9) in Python. Seurat integrates a full analytical pipeline covering normalization, dimensionality reduction, clustering, differential expression testing, and trajectory inference [204]. Its weighted nearest neighbor (WNN) algorithm enables efficient multi-modal data integration [205]. Dictionary-learning-based bridging integration aligns single-cell transcriptomic references with chromatin accessibility, DNA methylation, proteomic and other datasets, supporting ultra-large-scale analysis of tens of millions of cells [131] and offering novel strategies for cross-tissue and cross-developmental-stage integrated analysis in plants. For spatial omics data, Squidpy, built upon the Scanpy and AnnData ecosystem, adopts dual representations of spatial graphs and image containers to conduct in-depth spatial neighborhood analysis. It seamlessly interfaces with Napari for high-quality visualization and enables interactive exploration of large-scale spatial transcriptomic datasets [206]. Despite rapid advances, single-cell and spatial multi-omics data analysis still confronts persistent hurdles including data sparsity and batch effects [207].

5. Biological Validation and Breeding Application: Candidate functional SVs screened via multi-omics integrated analysis are further validated through gene editing, transgenic approaches, molecular marker development, and genomic selection for practical crop breeding [208,209,210].
  Recent pan-genome and SV research has shifted from traditional single-gene mapping to multi-dimensional integrated analysis, greatly boosting the practical breeding utility of plant SVs. For example, pan-genome SV analysis of wax apple successfully uncovers key causal genes controlling fruit size and cold tolerance [113]. In pepper, truncated conserved P-Y anchors in JAZ proteins reduce MeJA-triggered protein degradation, forming molecular brakes balancing plant growth and defense responses. The two divergent haplotypes of JAZ genes provide excellent genetic targets for climate-adaptive precision pepper breeding [114]. These practical applications fully demonstrate that SV-centered multi-omics analytical workflows provide powerful theoretical and technical support for modern molecular design breeding [211].
  Furthermore, plant genomics is undergoing a profound paradigm shift from single reference genomes to pan-genomes and 3D genomes. This transformation is primarily driven by rapid advances in LRS, spatial omics and multi-omics integration technologies, which enable unprecedented deep dissection of intraspecific genetic diversity, chromatin 3D architecture and cellular heterogeneity. As early as the start of this century, researchers have noted that genomics faces formidable challenges in massive data acquisition, storage, distribution and systematic analysis [212]. Accurately extracting biological insights from massive, multi-dimensional complex datasets remains a critical bottleneck restricting the development of plant functional genomics and molecular breeding [213].

### 3.4. Translating SV Knowledge into Breeding Practice

The ultimate goal of SV-centered genomics is to translate variant discovery into actionable breeding strategies [214]. Several complementary approaches can be employed:

Marker development and haplotype-based selection [215]: SVs that are consistently associated with agronomic traits can be converted into diagnostic molecular markers. Unlike SNP markers, SV-based markers (such as presence-absence variation markers or copy number variation markers) capture allelic diversity that is often inaccessible to SNP arrays [216]. Haplotype-resolved pan-genomes enable the identification of superior haplotypes that carry favorable SV combinations, facilitating marker-assisted selection and haplotype-based breeding [217].

Genomic prediction incorporating SV information: Traditional genomic prediction models rely primarily on SNP data. Recent studies have demonstrated that incorporating SV genotypes significantly improves prediction accuracy for complex traits [218], particularly for traits controlled by structural rearrangements such as inversions or large copy number variants. Graph pan-genomes provide a unified framework for integrating SNPs and SVs into prediction models, potentially increasing heritability capture and selection response [219].

Introgression breeding: Wild relatives of crops harbor abundant SVs associated with stress tolerance and disease resistance that are absent from elite cultivars. Pan-genome-guided introgression allows breeders to track the transfer of specific SV haplotypes from wild donors into cultivated backgrounds [157], minimizing linkage drag and accelerating pre-breeding programs [220].

Gene editing of SV loci: Once functionally useful SVs are cataloged, they can be introduced directly into elite breeding materials through gene editing rather than introgressed, transforming the pan-genome from a retrospective map into a forward-looking design toolkit. This strategy avoids linkage drag and accelerates the deployment of favorable SV haplotypes.

## 4. Challenges and Perspectives

**Figure 3:**
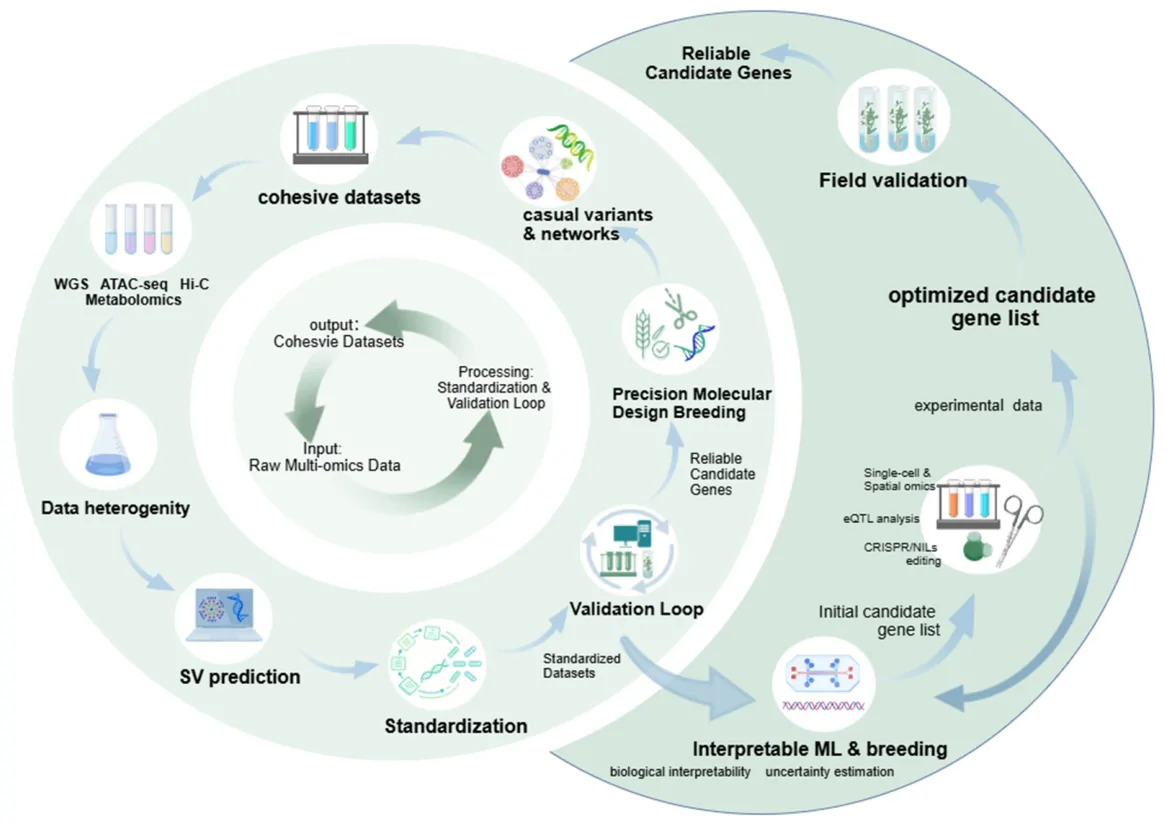
Schematic workflow of the analytical-experimental loop for converting raw plant multi-omics data into precision molecular-design breeding. This framework addresses key bottlenecks in plant multi-omics studies, including multi-omics data heterogeneity, inconsistent structural variant (SV) detection, and the black-box limitation of machine learning. Starting from raw multi-omics data such as WGS, ATAC-seq, Hi-C and metabolomics, fragmented datasets are standardized to eliminate batch effects and unify SV analysis. The core iterative standardization and validation loop integrates multi-omics evidence and functional assays including CRISPR-Cas9 editing and near-isogenic line characterization to verify bioinformatic predictions. Validated high-confidence candidate genes derived from interpretable machine learning and field validation support precision breeding, with updated empirical data further optimizing multi-omics resources to close the research cycle.

Despite remarkable progress in sequencing technologies, bioinformatic tools still face significant bottlenecks in deciphering plant genomic variants. Current challenges stem from the discordance among structural variant (SV) callers, the heterogeneity of multi-omics data formats, and the “black box” nature of machine learning applications in plant biology [178,221,222,223]. Machine learning opens new avenues for multi-omics integration and SV functional effect prediction, yet its application in plant systems biology still encounters obstacles [224]. Addressing these issues requires a paradigm shift from mere data accumulation to biologically meaningful integration (Figure 3). Following the hierarchical regulatory chain ranging from SV occurrence, through genomic consequences, regulatory effects and molecular changes, to cellular phenotypes and ultimately agronomic traits, this section systematically elaborates the current limitations, core challenges, and future frameworks for the functional interpretation of plant SVs [1] by layering multi-omics technologies [225,226], plant-specific genomic characteristics, and causal verification systems.

### 4.1. Genomics and Transcriptomics: Decoding Linear Regulatory Effects of SVs

Genomic and transcriptomic data constitute the foundational layers for identifying SV occurrence and dissecting its primary regulatory effects on gene transcription. The lack of unified standards for sample collection, quality control, and metadata annotation across laboratories introduces technical biases that obscure true biological signals underlying SV-gene expression associations. Notably, discordance among SV callers remains a critical pain point—integrative consensus strategies (e.g., VISTA frameworks) have proven effective in improving precision and F1 scores [178], yet such ensemble optimization approaches are far from routine practice in plant genomic research.

Transcriptomic profiling further bridges genomic SVs to molecular-level expression changes. eQTL analysis can distinguish cis- and trans-regulatory loci affected by SVs and link transcriptional variation to complex agronomic traits [187,227], while ASE in hybrid and polyploid plants directly detects parental expression bias, providing direct molecular evidence for SV-induced cis-regulatory variation [228]. To eliminate data fragmentation and technical heterogeneity in genomic and transcriptomic datasets, future efforts must prioritize the establishment of standardized multi-omics cohorts encompassing diverse cultivars, tissues, developmental stages, and environmental gradients. Developing reusable criteria for SV representation and data sharing will facilitate large-scale validation of genotype-environment-phenotype associations, transforming fragmented datasets into cohesive resources for the community [229].

### 4.2. Epigenomics and 3D Genome: Uncovering Chromatin-Level Genomic Consequences

Beyond linear genomic and transcriptomic variation, epigenomic modification and three-dimensional genome architecture dominate the higher-order genomic consequences and distal regulatory effects [230] of SVs. Structural variants do not merely alter linear DNA sequences or gene dosage; they can reshape chromatin neighborhood structures, remodel enhancer-promoter interaction patterns, and alter genome-wide chromatin accessibility and epigenetic modification landscapes, thereby triggering cascading regulatory changes in gene expression [186].

Current plant SV research often neglects the coupling relationship between SV breakpoints, 3D chromatin conformation, and epigenetic signatures. The integration of Hi-C, ChIP-seq, ATAC-seq, and BS-seq data enables systematic interpretation [231] of the complete regulatory cascade of “genetic variant-chromatin conformation-epigenetic state-gene expression” [186]. However, the heterogeneity of multi-omics data formats and inconsistent analysis pipelines seriously hinder the comprehensive elucidation of SV-mediated 3D regulatory mechanisms [178,221,222,223]. Standardized analytical frameworks for integrating 3D genome and epigenomic data are urgently needed [210] to clarify how different types of SVs reshape hierarchical chromatin structures and epigenetic modification patterns, and further mediate trait variation.

### 4.3. Proteomic and Metabolomic Layers: Linking Molecular Changes to Biochemical Phenotypes

Transcriptional variation induced by SVs cannot fully represent functional biological outputs, and proteomic and metabolomic analyses serve as key intermediate layers connecting SV-mediated molecular changes to macroscopic phenotypic formation [210,232,233]. Quantitative proteomics characterizes post-transcriptional regulatory events including protein translation efficiency, post-translational modification, and protein degradation turnover, while metabolomic profiling directly captures terminal small-molecule metabolic products [234,235] in plants.

Combined transcriptome-proteome-metabolome integration can effectively distinguish meaningless transcriptional noise from functional molecular alterations, pinpoint key biochemical bottlenecks caused by SVs, and clarify the core molecular mechanisms by which SVs drive phenotypic differentiation [236]. Nevertheless, the inconsistent detection platforms, data normalization standards, and annotation systems of proteomic and metabolomic data lead to severe inter-experimental heterogeneity [210,237], which greatly limits the accuracy and reproducibility of SV functional interpretation [221,222,223]. Optimized cross-omics unified analytical pipelines are required to realize the systematic parsing of SV regulatory effects from gene transcription to protein function and metabolic phenotype.

### 4.4. Single-Cell and Spatial Transcriptomics: Resolving Cell-Type-Specific Cellular Phenotypes

Traditional bulk multi-omics analyses average signals across mixed cell populations, masking cell-type-specific regulatory effects and cellular phenotypic changes induced by SVs. The rapid development of single-cell transcriptomics and spatial transcriptomics provides unprecedented high-resolution tools to break through this limitation and resolve SV-governed cellular phenotypes at microscopic resolution [238].

snRNA-seq avoids the technical obstacles of plant protoplast isolation and enables high-throughput profiling of cell-type-specific gene expression. Spatial transcriptomics represented by Stereo-seq preserves intact tissue spatial coordinate information, overcoming the spatial signal loss of conventional bulk RNA-seq [239]. The convergence of single-cell transcriptomics, spatial omics, and long-read haplotype-resolved assemblies enables researchers to unveil cell-type-specific regulatory effects of SVs that are previously masked by bulk tissue averaging, establishing a direct link between SV genetic variation, cell-level molecular changes, and tissue-level phenotypic differentiation. This cell-resolution analytical system fills the gap between molecular regulation and macroscopic agronomic traits, forming a complete hierarchical regulatory chain of SV function [240].

### 4.5. Challenges and Future Perspectives in SV Detection and Functional Interpretation in Plant Genomes

Different from animal and microbial genomes, plant genomes possess unique biological characteristics that bring persistent and specific challenges to SV detection and functional interpretation, including widespread polyploidy, highly repetitive genomic sequences, tissue heterogeneity, and strong environmental plasticity. First, most major crops are polyploid with complex subgenome homology and abundant homoeologous sequences, which easily lead to misalignment and false-positive/false-negative SV calls, severely interfering with accurate SV identification and haplotype differentiation [241]. Second, plant genomes are enriched with TEs and tandem repetitive sequences, which are core hotspots of SV formation but also the most difficult regions for sequencing assembly and variant detection, resulting in massive hidden SVs that cannot be captured by conventional pipelines [242]. Third, plant tissues exhibit significant cellular and developmental heterogeneity, and SV regulatory effects show obvious tissue-specific and spatiotemporal specificity, which cannot be fully resolved by traditional bulk sequencing strategies [233]. Fourth, plant traits have strong environmental plasticity; SV-genotype-phenotype associations are easily disturbed by environmental fluctuations, increasing the difficulty of stably identifying functional SVs [243]. In addition to plant inherent genomic characteristics, universal technical bottlenecks remain: persistent discordance among different SV callers, widespread multi-omics data format heterogeneity, and the “black box” nature of machine learning applications in plant biology [178,221,222,223]. Although machine learning opens new avenues for multi-omics integration and SV functional effect prediction, its application in plant systems biology still encounters substantial obstacles in interpretability and practical applicability [224].

### 4.6. From Statistical Association to Rigorous Causal Interpretation

Current plant SV research largely relies on statistical correlation analysis between variants and traits, while causal verification of SV function remains insufficient, forming a critical gap in the regulatory chain from molecular variation to agronomic traits. Computational predictions regarding SVs and their downstream regulatory elements require rigorous experimental validation to establish definite causal relationships rather than passive statistical association.

An integrated multi-level validation pipeline should be constructed to assemble cumulative evidence: first, preliminary screening and functional inference based on eQTL analysis, ASE profiling, chromatin conformation capture, and multi-layer epigenetic profiling; second, strict functional verification via CRISPR-Cas9 gene editing, transgenic complementation experiments, and near-isogenic line population validation, which is indispensable to confirm the precise impact of specific SVs on molecular regulatory networks and macroscopic agronomic phenotypes. This step-by-step verification system fundamentally solves the problem of confusing correlation with causation in current SV research, realizing the transformation from statistical association to biological causal interpretation [244].

### 4.7. Integrated Conceptual Framework for SV Interpretation and Molecular Breeding

To address the above challenges, future plant SV research requires a unified, interpretable, and breeding-oriented integrated conceptual framework, realizing the paradigm shift from passive data accumulation to active biologically meaningful integration. While machine learning opens new avenues for SV functional prediction, reliance on predictive accuracy alone is insufficient for practical breeding applications. The utility of these models must be comprehensively evaluated based on biological interpretability, cross-population robustness, and field breeding practicability.

Future intelligent analytical models should fully integrate multi-dimensional information including genetic variants, multi-omics molecular regulatory networks, environmental variables, and high-throughput phenotypic data, and incorporate uncertainty estimation to improve prediction reliability. Validating these models in independent germplasm and field conditions will enhance the reliability of candidate gene prioritization, steadily translating genomic discoveries into precise molecular design breeding strategies [229,245]. Ultimately, this layered multi-omics framework, combined with causal verification systems and interpretable machine learning models, can steadily transform basic genomic discoveries into precise molecular design breeding strategies, facilitating the efficient utilization of SV genetic resources for crop improvement and agricultural innovation [246].

The practical implementation of SV-based breeding strategies depends not only on scientific advances but also on the accessibility and cost-effectiveness of different sequencing technologies. While PacBio HiFi provides high-accuracy assemblies, its per-genome cost remains substantially higher than Illumina short-read sequencing. Oxford Nanopore offers lower initial investment and portable devices suitable for field-based applications, but its higher error rate requires careful bioinformatic handling. For breeding programs, a tiered approach may be optimal: short-read resequencing for population-scale genotyping, combined with targeted long-read validation for key genomic regions identified as SV hotspots. Future cost reductions and the development of user-friendly analysis pipelines will be critical for translating SV knowledge into routine breeding practices, particularly in developing countries and for understudied orphan crops.

## 5. Conclusions

Structural variations have emerged as core drivers of plant genome plasticity, shaping adaptive evolution and agronomic diversity through TE activity, NAHR, HGT, and polyploidization-associated restructuring. Long-read sequencing and graph-based pan-genomes have largely overcome the limitations of linear reference genomes, enabling unbiased SV discovery and genotyping. Concurrently, multi-omics integration—spanning genomics, transcriptomics, epigenomics, 3D chromatin, proteomics, metabolomics, and spatial transcriptomics—provides a layered framework linking SVs to regulation and phenotypes from molecular to cellular levels.

Nevertheless, critical bottlenecks persist. Discordance among SV callers, multi-omics data heterogeneity, and the “black-box” nature of machine-learning models undermine reproducibility, while plant-specific challenges (polyploidy, repetitive sequences, environmental plasticity) complicate detection and inference. Closing the gap from statistical association to causal validation remains essential. Future priorities should include standardized multi-omics cohorts, interpretable and robust computational models, and translation of SV knowledge into breeding pipelines—with particular attention to cost-effective, user-friendly workflows accessible to understudied crops and resource-limited regions. By bridging genomic variation with functional phenotypes, SV-centered genomics holds transformative potential for precision crop improvement.

## Figures and Tables

**Table 1:** Summary of Recent Representative Studies on SVs in Plants.

Species	SV Types	Detection Method	Functional Impact
*Solanum lycopersicum*	deletion	LRS	fruit quality regulation [105].
*Brassica juncea*	deletion	genome comparison	trichome development [106].
*Pyrus* spp.	14 bp deletion; nonsense mutation	BSA-seq, QTL, WGR	anthocyanin and dwarfism regulation [107].
*E. grandis* × *E. urophylla* F_1_ hybrid	Inversion, translocation, duplication, deletion, insertion	Nanopore LRS + trio-binning + SyRI	Genome structural variation; candidate basis of hybrid superiority [108].
*Sorghum bicolor*	Deletion, insertion, inversion	pan-genome analysis	stress adaptation [24,109]
*Brassica rapa*	PAV	pangenome analysis	disease resistance regulation [110]
*Gossypium purpurascens*	inversions	population genomics	fiber quality [111]
*Helianthus* spp.	inversion, deletion, gene PAV, translocation	LRS, Hi-C, genome comparison	suppress recombination; drive adaptation and speciation; modulate agronomic traits and heterosis [112]

**Table 2:** Representative Applications of Single-Cell and Spatial Transcriptomics in Plant Research.

Application	Representative Finding
Mechanisms of Root Development and Lateral Root Formation	(1) Single-cell RNA sequencing (scRNA-seq) in *Arabidopsis* uncovers lateral root primordium (LRP) cell origin: pericycle precursor cells reprogram into mixed ground tissue/stem cell niche and vascular progenitor populations, then differentiate into specialized phloem cells for sucrose transport [196]. (2) ScRNA-seq of 3121 *Arabidopsis* root cells reconstructs pseudotime trajectories, identifies hundreds of stage-specific genes; transcription factor motifs enriched in early/late cells drive root cell fate branching [197]. (3) ScRNA-seq of 12,172 sweet potato root tip cells identifies 15 cell clusters; transcription factor IbGATA4 regulates adventitious root development [198].
Floral Organ Development and Spatial Transcriptome Reconstruction	(1) Single-nucleus RNA-seq (snRNA-seq) with spatial reconstruction maps 3D gene expression in *Arabidopsis* floral meristems, revealing spatiotemporal vascular initiation for organ patterning [199]. (2) snRNA-seq uncovers stage-specific transcription factors and cell-type regulatory networks during early anther development [200].
Stress Response and Cell-Type-Specific Defense Mechanisms	(1) ScRNA-seq of 29,217 maize root tip cells identifies distinct responses of seven cell types to *Fusarium verticillioides*, constructs an immune network with 4049 differentially expressed genes (DEGs) and predicts 42 disease-resistant QTLs [201].
Nutrient Stress and Cellular Adaptation	(1) Pea scRNA-seq under boron deficiency shows repressed photosynthetic genes in mesophyll, reduced guard cell stomatal density, and downregulated chromatin remodeling genes in the shoot apical meristem (SAM) [202].

## Data Availability

No new data were created or analyzed in this study.

## Abbreviations

ASE	Allele-Specific Expression
ATAC-seq	Assay for Transposase-Accessible Chromatin using sequencing
BS-seq	Bisulfite Sequencing
BSA-seq	Bulked Segregant Analysis sequencing
CCS	Circular Consensus Sequencing
ChIA-PET	Chromatin Interaction Analysis by Paired-End Tag Sequencing
ChIP-seq	Chromatin Immunoprecipitation Sequencing
CNVs	Copy Number Variations
CTs	Chromosome Territories
DEGs	Differentially Expressed Genes
eQTL	Expression Quantitative Trait Locus
HGT	Horizontal Gene Transfer
indels	Insertions/Deletions
JAZs	Jasmonate ZIM-domain proteins
LRP	Lateral Root Primordium
LRS	Long-Read Sequencing
LTR	Long Terminal Repeat
MeJA	Methyl Jasmonate
MITEs	Miniature Inverted-Repeat Transposable Elements
NAHR	Non-allelic Homologous Recombination
NBS-LRR	Nucleotide-Binding Site—Leucine-Rich Repeat
NGS	Next-Generation Sequencing
NHEJ	Non-homologous End Joining
ONT	Oxford Nanopore Technologies
PAV	Presence-absence variation
PAVs	Presence–absence variations
P-Y	Proline-Tyrosine
QTL	Quantitative Trait Locus
SAM	Shoot Apical Meristem
R genes	Resistance genes
RNA-seq	RNA Sequencing
SBS	Sequencing-By-Synthesis
scRNA-seq	Single-Cell RNA Sequencing
SMRT	Single Molecule, Real-Time
SNPs	Single Nucleotide Polymorphisms
snRNA-seq	Single-nucleus RNA-seq
SV	Structural Variation
SVs	Structural Variations
SyRI	Synteny and Rearrangement Identifier
T2T	Telomere-to-Telomere
TADs	Topologically Associating Domains
TEs	Transposable Elements
TGS	Third-Generation Sequencing
WGCNA	Weighted Gene Co-expression Network Analysis
WGD	Whole-genome duplication
WGR	Whole-Genome Resequencing
WGS	Whole-Genome Sequencing
WNN	Weighted Nearest Neighbor
XGBoost	eXtreme Gradient Boosting
